# Cue-Elicited Brain Activity and Treatment Outcomes in Substance Use Disorders: A Meta-Analysis

**DOI:** 10.1001/jamanetworkopen.2025.48809

**Published:** 2025-12-11

**Authors:** Bryn Evohr, Sean Wenzel, Nicole Chen, Carrie Wade, Hamed Ekhtiari, Adam C. Ketron, Amy C. Janes, Jodi M. Gilman

**Affiliations:** 1 Center for Addiction Medicine, Department of Psychiatry, Massachusetts General Hospital, Boston; 2 Countway Library of Medicine, Harvard Medical School, Boston, Massachusetts; 3 Department of Psychiatry, University of Minnesota, Minneapolis; 4 National Institute on Drug Abuse, Intramural Research Program, Baltimore, Maryland; 5 Department of Psychiatry, Harvard Medical School, Boston, Massachusetts

## Abstract

**Importance:**

Substance use disorders (SUDs) affect millions of of people worldwide and are marked by high relapse rates. Cue-elicited brain activity may correlate with treatment success, but no meta-analysis has identified which brain regions are consistently associated with outcomes.

**Objective:**

To evaluate, across the existing literature, whether functional magnetic resonance imaging (fMRI)–based cue reactivity is associated with SUD treatment response (eg, predictive biomarker) or posttreatment neural changes (eg, response biomarker).

**Data Sources:**

A systematic search of PubMed, PsycINFO, Embase, and Cochrane databases covered studies published from database inception through May 2023.

**Study Selection:**

Studies were included if they reported fMRI-based cue reactivity and treatment outcomes for adults undergoing SUD treatment.

**Data Extraction and Synthesis:**

Data extraction followed PRISMA guidelines. Analyses were conducted from October 2024 to January 2025 using Seed-Based *d* Mapping With Permutation of Subject Images to quantify the magnitude and extent of activation to substance-related cues across studies. Random-effects models were used to generate statistical activation maps.

**Main Outcomes and Measures:**

Statistical maps of cue-induced activation during drug cue presentation at baseline correlating with a treatment outcome (for predictive biomarkers) as well as in brain regions exhibiting a significant change after treatment (for response biomarkers). Statistical inference used permutation statistics, with thresholds set at *P* < .005 and cluster extent of 10 voxels.

**Results:**

Fifty-one studies (1787 participants) were included. Cue-elicited activation was observed across studies in the cingulate gyrus, middle frontal gyrus, caudate, and insula. Predictive biomarker regions (n = 14 studies, 456 participants) included the bilateral insula and cingulate, in which lower baseline cue reactivity was associated with better treatment outcomes. Response biomarkers (pretreatment to posttreatment analyses; n = 25 studies, 609 participants) indicated significant reductions in cue reactivity in the anterior cingulate, caudate, accumbens, and insula. Cue-elicited activation in the insula emerged as both predictive and response biomarkers.

**Conclusions and Relevance:**

This meta-analysis of cue-induced brain activation in adults with SUDs identified specific regions consistently engaged during cue exposure that may serve as candidate predictive and response biomarkers for treatment outcomes, highlighting the potential role of brain-based biomarkers in informing interventions for SUD.

## Key Points

**Question:**

Can functional magnetic resonance imaging (fMRI) cue reactivity serve as a predictive biomarker or response biomarker of treatment outcomes in individuals with substance use disorders?

**Findings:**

This meta-analysis of 51 fMRI cue reactivity studies with 1757 participants identified consistent cue-elicited brain activation patterns. Lower baseline activation in the insula and cingulate was associated with better treatment outcomes, whereas regions including the cingulate, caudate, accumbens, and insula, showed reduced activation following treatment.

**Meaning:**

fMRI cue reactivity shows promise as a predictive biomarker and response biomarker for substance use disorder treatment, supporting its potential utility in guiding and evaluating therapeutic interventions.

## Teaser

This meta-analysis evaluates whether treatment response is associated with cue reactivity in key neural regions among patients being treated for substance use disorder.

## Introduction

Substance use disorders (SUDs) represent a substantial global public health challenge. The lifetime prevalence of SUDs is estimated at 35%, leading to approximately 11.8 million deaths each year.^1^ Additionally, SUDs impose considerable societal and economic costs, with annual expenses in the US exceeding $740 billion.^2^ Despite advancements in treatment methods, relapse rates remain high, ranging from 40% to 60%^3^ and highlighting the need for a better understanding of the mechanisms that drive drug-seeking.

Cue reactivity—a phenomenon in which drug-related cues provoke craving, emotional arousal, and physiologic responses—is a critical driver of relapse.^4^ Over time, cues present during drug use become linked with the drugs and related behaviors, leading to the formation of conditioned responses to the cues themselves.^5^ Preclinical research has clearly shown that exposure to drug-associated cues markedly increases drug-seeking behavior and consumption.^6,7^ Exposure to drug-related cues similarly triggers drug-seeking behavior in humans.^8^ A previous systematic review and meta-analysis indicated that self-reported cue-induced craving played significant roles in drug use and relapse outcomes, making these important mechanisms underlying SUDs.^9^

Investigating how the brain processes such cues is essential for designing effective interventions.^10^ Functional magnetic resonance imaging (fMRI) studies have played a critical role in elucidating the neural underpinnings of cue reactivity, revealing activation in regions such as the prefrontal cortex, anterior cingulate cortex, amygdala, insula, and striatum.^4,11,12^ Understanding cue reactivity at the neural level has implications for personalized treatment because biomarkers derived from cue reactivity paradigms could inform intervention development or clinical care for individuals with SUDs.^13^ For instance, interventions such as cognitive behavioral therapy,^14^ mindfulness-based approaches,^15^ and pharmacotherapies such as varenicline^16^ have shown modulation of cue-induced brain activation. This modulation could serve as a potential biomarker that underlies the effectiveness of the intervention.

Recent efforts have focused on evaluating cue-induced neural activity, measured via fMRI, as a potential biomarker for estimating treatment outcomes in SUDs. According to US Food and Drug Administration definitions,^17^ a predictive biomarker is a baseline measure that identifies individuals more likely to benefit from a particular treatment, whereas a response biomarker indicates a biological change after treatment, reflecting that the intervention has engaged its intended target or mechanism. No studies have systematically assessed, across the literature, whether there are consistent patterns of cue-induced brain biomarkers in individuals undergoing treatment for SUDs. Other meta-analyses of cue reactivity fMRI studies have demonstrated consistent networks of brain regions responsive to drug cues, including brain networks involved in reward processing, attention, and emotional regulation, but existing meta-analyses typically identify regions activated by drug cues rather than examine whether cue reactivity is associated with treatment outcomes^18,19,20^ or focus on only one substance (eg, alcohol).^21^ The current meta-analysis addresses this gap by identifying reliable and convergent neural responses to drug cues specifically across treatment studies and, uniquely, by separating studies that assess cue reactivity at baseline (predictive biomarkers) from those examining changes before to after treatment (response biomarkers), allowing for a more precise evaluation of how cue-induced brain activity may function as a clinically meaningful biomarker in different phases of treatment. By synthesizing findings across studies, we aimed to identify key neural regions that underlie cue reactivity and show promise for guiding treatment strategies.

## Methods

### Search and Inclusion of Studies

**Figure 1.**
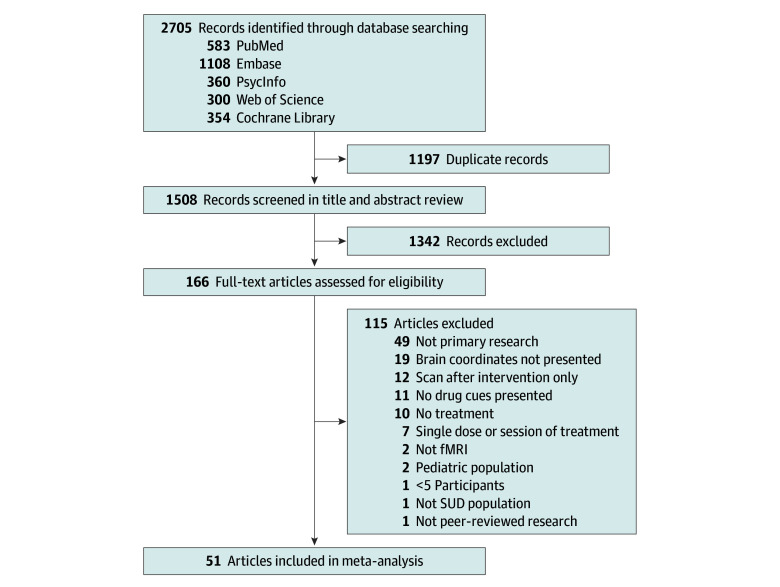
PRISMA Diagram fMRI indicates functional magnetic resonance imaging; SUD, substance use disorder.

This review adheres to the Preferred Reporting Items for Systematic Reviews and Meta-Analyses (PRISMA) guidelines.^22^ The protocol was registered with the Prospective Register of Systematic Reviews (PROSPERO) (CRD42023435296). Electronic databases, including PubMed, PsycInfo, Embase, Web of Science, and Cochrane, were searched on May 24, 2023, for fMRI cue reactivity studies involving adults with SUDs undergoing treatment (see eMethods in Supplement 1 for complete search terms). As described in the PRISMA flow diagram (Figure 1), studies included at the title and abstract screening phase were those that assessed adults with SUDs undergoing treatment and presented drug and neutral cues during fMRI scanning before treatment was initiated. Studies were excluded if they were not peer-reviewed, did not collect original data, or did not include human adult participants. During the full-text review, inclusion criteria required studies to include neuroimaging scans with cue reactivity paradigms collected before treatment, assess SUD outcomes after treatment, and sample at least 5 individuals. Studies were excluded if they did not provide peak coordinates in MNI (Montreal Neurological Institute) or Talairach space. Additional details (peak coordinates or statistical maps) were obtained from the study authors whenever necessary.

During the sequential screening process (title and abstract screening as well as full-text review), 2 authors (B.E., S.W., N.C., A.C.K., and/or J.M.G.) independently assessed potential articles. All conflicts were resolved through consensus, resulting in moderate interrater reliability of 0.42 during abstract screening and good interrater reliability of 0.60 to 0.80 during full-text review, as determined by the Cohen κ.

### Data Extraction

Relevant and available data from all included studies were extracted, including study characteristics (eg, authors, publication year, and sample size), participant attributes (eg, age, gender, and clinical characteristics), details of the experimental design (eg, task type, duration, and stimuli), imaging parameters (eg, scanner type and acquisition sequence), and outcome measures (eg, brain regions of interest, statistical values, and substance use outcomes). One author (S.W. or N.C.) completed the data extraction, whereas another (B.E. or J.M.G.) verified all extracted values.

### Statistical Analysis

Our primary outcome of interest was *z* scores of brain activation in response to drug cues (compared with neutral cues). When *z* scores were not presented, we converted *t* statistics or *P* values to *z* scores for analysis. Voxel-wise meta-analyses of regional differences in brain activity were conducted using the Seed-Based *d* Mapping With Permutation of Subject Images (SDM-PSI) software package, version 6.23 (SDM Project).^23^ SDM-PSI processes peak brain coordinates with an anisotropic unnormalized gaussian kernel to generate statistical parametric maps of effect sizes and variance. It then calculates the mean of voxel values across the included studies, weighted by the inverse of the variance and accounting for interstudy heterogeneity.

We conducted an omnibus meta-analysis on all included studies, examining the differential brain activation in response to drug cues vs neutral cues (Table). We then performed meta-analyses on specific categories of studies. To evaluate cue reactivity as a predictive biomarker, we analyzed studies reporting cue-induced activation measured before treatment statistically associated with a future clinical outcome (eg, abstinence, relapse, craving and reduction). Outcomes included both direct and proxy measures of drug use, such as drug use frequency, drug use or relapse latency, and relapse drug use in individuals who had previously been abstinent. To evaluate cue reactivity as a response biomarker, we completed 2 additional meta-analyses. First, we assessed studies reporting a within-participant change in cue-induced activation from baseline (pretreatment) to follow-up (posttreatment), referred to as *time*. Second, we assessed studies reporting significant interactions between time (pretreatment vs posttreatment) and treatment group (active vs control), referred to as time × treatment. Lastly, we completed subgroup analyses assessing studies by primary substance (alcohol and nicotine) and by treatment type (pharmacologic and psychosocial); there were insufficient numbers of studies involving other substance use populations or involving brain-based (real-time fMRI and transcranial magnetic stimulation) treatments for subgroup analyses.

**Table.** Summary Characteristics of the Included Studies

Measure	Omnibus	Predictive biomarker^a^	Response biomarker (time)^b^	Response biomarker (time × treatment)^c^
No. of studies	51	14	19	13
No. of participants	1787	456	374	380
Age, mean (SD), y	39 (9.3)	34.8 (9)	42.3 (9.4)	39.5 (8.4)
Sex, mean (SD), %				
Female	34.9 (26.3)	35.7 (29.4)	34.6 (31.6)	24.1 (24.2)
Male	65.1 (26.3)	64.3 (29.4)	65.4 (31.6)	75.9 (24.2)
Treatment type, No. (%)^d^				
Pharmacologic	33 (65)	10 (62)	13 (68)	7 (54)
Psychosocial	24 (47)	8 (50)	7 (37)	5 (38)
Circuit based	5 (10)	3 (19)	3 (16)	2 (15)
Primary substance, No. (%)				
Alcohol	24 (47)	5 (31)	10 (53)	7 (54)
Nicotine	13 (25)	5 (31)	3 (16)	4 (31)
Stimulants	6 (12)	2 (12)	1 (5)	0
Opioids	5 (10)	2 (12)	3 (16)	0
Cannabis	2 (4)	2 (12)	1 (5)	2 (15)
Polysubstance	1 (2)	0	1 (5)	0

^a^ Studies reporting cue-induced activation measured before treatment that is statistically associated with a future clinical outcome (eg, abstinence, relapse, and craving reduction).

^b^ Studies reporting within-participant changes in cue-induced activation from baseline (pretreatment) to follow-up (posttreatment).

^c^ Studies reporting significant interactions between time (pretreatment vs posttreatment) and treatment group (active vs control).

^d^ Numbers total greater than 100% because several studies used multiple treatment modalities.

Statistical significance was established according to the recommended threshold of uncorrected, 2-sided *P* < .005, SDM-PSI *z* > 1, and a cluster extent of 10 voxels.^24^ Familywise error correction was also evaluated at *P* = .05 with a cluster extent of 10 voxels to identify highly significant clusters, using threshold-free cluster enhancement statistics.^25^ Extracted data were processed and prepared for analysis in R software, version 4.4.0 (R Foundation for Statistical Computing). We also assessed heterogeneity with the *I*^2^ index (the proportion of variability in voxel-wise effect sizes that is due to true differences among studies), publication bias with the metabias *P* value (a statistical measure used to detect potential publication bias or small-study effects), and excess significance *P* value (also a measure of potential reporting bias or inflation of effects).

## Results

### Included Studies and Sample Characteristics

Of 1508 abstracts (after removing duplicates), 166 studies were screened for full-text criteria, and 51 studies^26,27,28,29,30,31,32,33,34,35,36,37,38,39,40,41,42,43,44,45,46,47,48,49,50,51,52,53,54,55,56,57,58,59,60,61,62,63,64,65,66,67,68,69,70,71,72,73,74,75,76^ were included in the omnibus meta-analysis (Figure 1). Summary characteristics of the studies are presented in Table. eTables 1 and 2 in Supplement 1 provide more information about the methods and demographics of each study included in the meta-analyses and a complete list of studies included in each independent meta-analysis. These 51 studies include 32 randomized clinical trials,^27,29,30,31,33,34,35,36,37,38,45,46,47,48,49,50,55,56,57,58,60,61,62,63,65,66,67,69,71,73,74,75^ 15 nonrandomized clinical trials,^26,28,32,39,40,41,42,43,44,54,64,68,70,72,76^ and 4 cohort studies.^51,52,53,59^ Treatments spanned pharmacologic agents (n = 27 studies),^27,30,31,32,33,36,37,38,39,42,43,47,48,49,51,52,53,55,57,58,60,65,66,67,70,72,73^ behavioral therapy or psychotherapy (n = 13),^28,29,34,35,54,59,61,62,68,71,74,75,76^ neuromodulation (n = 5),^40,41,45,46,63^ and combined pharmacologic and behavioral approaches (n = 6).^26,44,50,56,64,69^ The majority of studies focused on alcohol (n = 24)^26,27,28,30,32,35,36,37,38,40,41,42,45,48,49,55,56,60,67,68,71,73,74,75^ and nicotine (n = 15),^29,31,34,39,43,44,46,47,56,63,64,76^ with smaller groups investigating opioids (n = 5),^51,52,53,70,72^ stimulants (n = 6),^50,59,62,65,66,69^ cannabis (n = 2),^57,58^ and polysubstance use (n = 1).^61^

### Brain Activation in Response to Drug Cues 

**Figure 2.**
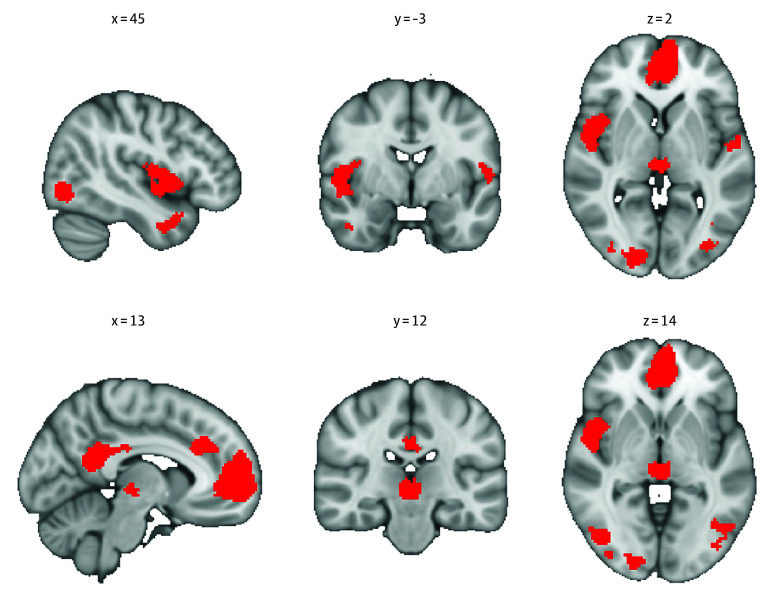
Brain Activation to Drug Cues Over Neutral in Omnibus Meta-Analysis Red color indicates significant activation to drug cues compared with neutral cues.

In our omnibus meta-analysis of SUD treatment studies (n = 51), increased activation to drug vs neutral cues was observed in the paracingulate gyrus, anterior cingulate gyrus (*z* = 5.833; *P* < .001), and posterior cingulate gyrus (*z* = 5.864; *P* < .001), right insula (*z* = 4.83; *P* < .001), bilateral inferior temporal gyrus (right: *z* = 4.052 and left: *z* = 5.143; *P* < .001), right superior parietal gyrus (*z* = 3.809; *P* < .001), and thalamus (*z* = 4.835; *P* < .001) (Figure 2; eFigure 1 and eTable 3 in Supplement 1). Neutral cues never elicited greater activation than drug cues.

### Predictive Biomarkers

We next examined studies that presented baseline brain activation to drug cues that were correlated with future substance use outcomes after treatment (n = 14 studies).^28,33,35,39,40,44,45,46,50,57,58,64,65,72^ Sample sizes ranged from 17 to 87 participants, and studies spanned a wide range of substances, including alcohol, nicotine, opioids, stimulants, and cannabis. Treatments investigated included behavioral counseling, varenicline, nicotine replacement therapy, methadone maintenance, pharmacotherapies such as sertraline and naltrexone, and cognitive-behavioral interventions (eTable 2 in Supplement 1). Across these studies, we found that lower baseline cue-induced activation in the bilateral insula (right: *z* = −3.469 and left: *z* = −3.559; *P* < .001), bilateral midcingulate and paracingulate gyri (left: *z* = −2.771; *P* = .003; and right: −3.185; *P* < .001), and precuneus (*z* = −3.007; *P* = .001) was associated with better treatment outcomes (eFigure 2 and eTable 4 in Supplement 1). In contrast, greater activation in the subgenual anterior cingulate cortex (*z* = 3.035; *P* = .001) correlated with better treatment outcomes.

### Response Biomarkers

For response biomarkers, sample sizes ranged from 10 to 90 participants. Studies included alcohol, nicotine, cannabis, stimulants, opioids, and polysubstance use. Interventions were diverse, including pharmacotherapies (eg, naltrexone, baclofen, topiramate, clozapine, and risperidone), neuromodulation approaches (eg, transcranial magnetic stimulation, transcranial direct current stimulation, and neurofeedback), and psychosocial or cognitive therapies (eg, cue exposure, mindfulness-based therapy, and cognitive bias modification) (eTable 2 in Supplement 1).

In our first analysis (time), we examined studies with at least 2 cue reactivity scans: one before treatment and another during or after treatment (n = 19 studies).^26,27,31,32,39,40,41,43,45,48,49,51,52,57,61,66,70,74,75^ We observed decreases in brain activation to drug cues after treatment in regions such as the right olfactory cortex (*z* = −4.283; *P* < .001), left amygdala (*z* = −4.335; *P* < .001), left insula (*z* = −3.308; *P* < .001), left parahippocampal gyrus (*z* = −3.686; *P* < .001), and right putamen (*z* = −3.343; *P* < .001) (eFigure 3 and eTable 5 in Supplement 2).

In a second analysis (time × treatment analysis), we examined studies reporting significant interactions between time (pretreatment vs posttreatment) and treatment group (active vs control) (n = 13 studies).^26,27,31,34,41,47,57,58,63,71,73,74,75^ Cue-elicited brain activation decreased more over time in individuals receiving treatment than in those receiving a placebo in the right temporal pole (*z* = −4.413; *P* < .001), left striatum (*z* = −3.389; *P* < .001), bilateral thalamus (*z* = −3.069; *P* = .001), bilateral insula (right: *z* = −3.302 and left: −3.294; *P* < .001), and frontal regions (*z* = −3.458; *P* < .001) (eFigure 4 and eTable 6 in Supplement 1).

### Predictive and Response Biomarker Overlap

**Figure 3.**
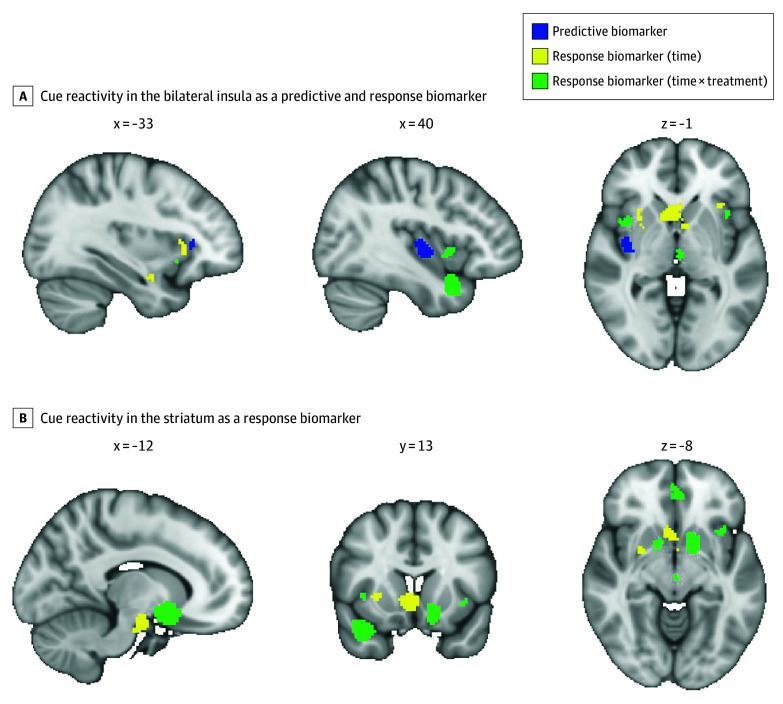
Cue Reactivity as a Predictive and Response Biomarker

The bilateral insula was identified as both a predictive biomarker, where decreased cue reactivity estimated that individuals were more likely to experience better treatment outcomes, and a response biomarker, where cue reactivity decreased in response to treatment (Figure 3A). In contrast, the striatum was identified specifically as a response biomarker, decreasing over time in response to treatment in all participants together and more in the active treatment group compared with those receiving placebo or less effective treatment (Figure 3B).

### Heterogeneity and Publication Bias

Heterogeneity was low across meta-analyses. All statistically significant brain regions in the predictive biomarker analysis and both response biomarker analyses reported low *I*^2^ statistics (<40%), indicating a low percentage of variance attributable to study heterogeneity^77^ (eTables 4-6 in Supplement 1). Similarly, most statistically significant brain regions in the omnibus meta-analysis reported low *I*^2^ statistics (eTable 3 in Supplement 1). Although no regions in the predictive biomarker and response biomarker meta-analyses demonstrated significant bias on the slope-based metabias test,^78^ these tests have limited sensitivity and may fail to detect bias when it manifests as truncations around significance thresholds rather than a linear association with study precision. Visual inspection of funnel plots revealed some asymmetries consistent with small-study effects (eFigure 5 in Supplement 1), and the excess significance test found potential reporting bias in 3 brain regions in the predictive biomarker meta-analysis (right and left paracingulate gyrus and left precuneus) and 1 brain region in the response biomarker meta-analysis (right putamen), as well as several regions in the omnibus meta-analysis, suggesting potential reporting bias (eTables 3-6 in Supplement 1).

### Sensitivity Analyses

Subgroups within the omnibus meta-analysis were analyzed where sample sizes were large enough. A subanalysis of alcohol studies found consistent brain activation to cue reactivity compared with the omnibus meta-analysis (eTable 7 in Supplement 1), and no significant clusters emerged from a subanalysis of nicotine studies. A subanalysis of studies using pharmacologic treatment identified brain activation to cue reactivity in the left insula, right precuneus, striatum, and frontal regions, and studies using psychosocial treatment identified activation similar with that in the omnibus meta-analysis (eTables 8 and 9 in Supplement 1).

## Discussion

This meta-analysis synthesizes findings from 51 studies involving 1787 participants to elucidate the association between fMRI-derived cue reactivity and treatment outcomes in adults receiving treatment for SUDs. Our meta-analysis identified consistent drug cue-induced activation in salience, cingulate, and temporal-parietal regions. Lower baseline activation in the insula and cingulate was associated with better treatment outcomes, supporting a potential role of cue-induced brain activity in these regions as a predictive biomarker. Treatment-related reductions in cue reactivity in the insula, amygdala, and thalamus were observed over time and were more pronounced in active treatment groups, consistent with a response biomarker pattern. These findings support potential utility of fMRI cue reactivity as both predictive and response biomarkers of treatment outcomes in SUDs.

The findings highlight the importance of a network of brain regions, including the anterior cingulate cortex, caudate, accumbens, and insula, in treating SUDs.^79^ Collectively, these regions are essential for reward processing, craving, cognitive control, and interoceptive awareness, all of which are vital to addiction and recovery.^6,18^ There was substantial overlap between predictive and response biomarkers, with the bilateral insula emerging as both: lower baseline activation was associated with better treatment outcomes, and activation decreased after treatment. The insula, which integrates interoceptive signals with emotional and cognitive processes, plays a critical role in craving and relapse risk.^80^ Lower posttreatment activity likely corresponds to diminished interoceptive awareness of drug-related urges, aligning with improved treatment outcomes. Other notable regions included the midcingulate and paracingulate cortices, components of the salience network^3,18,19^ where lower baseline cue-induced activation was associated with more favorable treatment outcomes. The significant reductions in neural cue reactivity across these regions align with previous research^7,15,16,81,82^ emphasizing the importance of cortico-limbic circuits in SUD risk and treatment. Reduced engagement of these circuits at baseline may reflect lower risk for relapse or greater capacity for treatment engagement.^18,20^

The striatum emerged specifically as a potential response biomarker, exhibiting significant reductions in cue-induced activation after treatment. This effect was observed both across all participants over time and more prominently in individuals receiving active treatment compared with those in placebo control conditions, suggesting that striatal reactivity is sensitive to therapeutic engagement and may reflect treatment-induced changes in motivational or reward processing.^6,9,12,81^ Treatment-related reductions in these areas may reflect normalization of reward circuitry. The thalamus, which serves as a relay for sensory and affective processing, and the parahippocampal gyrus, involved in contextual memory and cue associations,^7,83^ also showed cue reactivity reductions, suggesting a downregulation of memory-driven drug-seeking responses. Collectively, these changes suggest that effective treatment may reduce the heightened salience of drug-related cues. Importantly, several of these regions, particularly the dorsolateral and medial prefrontal cortex (including parts of the superior frontal gyrus and medial orbital cortex), are emerging targets for transcranial magnetic stimulation in the treatment of SUDs.^84^ These findings support the potential mechanistic relevance of these circuits and provide further rationale for neuromodulation-based interventions aimed at reducing cue-driven relapse risk.

This meta-analysis builds on previous research by integrating findings across various substances, including alcohol, nicotine, stimulants, opioids, and cannabis, to emphasize shared neural mechanisms of cue reactivity. The findings may have clinical implications. First, fMRI-derived measures of cue reactivity could serve as potential biomarkers for relapse risk and treatment success. Second, interventions targeting these regions, such as neurofeedback, cognitive behavioral therapy, or pharmacological agents, could potentially improve treatment efficacy by directly modulating the neural circuits involved in craving and reward processing. Third, integrating cue reactivity measures into clinical trials could enhance the evaluation of therapeutic interventions, particularly for assessing pharmacologic efficacy. The observed reductions in cue reactivity within the regions above suggest that effective interventions, whether pharmacologic or psychological, may not merely alleviate symptoms but also alter neural circuits that underlie addiction.^6,18^

### Limitations

This study has limitations. The identified biomarkers do not yet meet the strict definition of predictive, which requires showing that baseline neural measures predict treatment-specific benefit. Instead, our findings indicate that lower cue reactivity in regions such as the insula and cingulate is associated with better outcomes across heterogeneous treatment modalities. This distinction reflects the current state of the literature: most included trials were not designed to test biomarker × treatment interactions but rather to examine estimates of general treatment response. Future studies explicitly designed to evaluate biomarker-treatment matching are needed to determine whether the insula, cingulate, or striatal reactivity can serve as true predictive biomarkers for personalized interventions. Furthermore, given the evidence for small-study effects, excess significance, and possible publication bias, these findings should be regarded as hypothesis-generating rather than definitive.

An additional limitation is the lack of standardized effect size reporting across included neuroimaging studies. Results are often presented in terms of statistical significance or cluster extent rather than standardizing effect magnitude. This also prevented generation of canonical forest plots, which typically display per-study effect sizes, variances, and pooled estimates for each region of interest. Routine reporting of standardized effect sizes would strengthen reproducibility and the cumulative evidence base, and we encourage neuroimaging researchers to adopt this practice.

Furthermore, many included studies had small sample sizes, which limits the generalizability of the findings and raises the possibility of selective reporting, even when formal metabias tests are not statistically significant. Questions also remain about the test-retest reliability of fMRI signals.^85^ Inconsistencies in fMRI acquisition parameters and analytical approaches further contribute to variability in reported neural responses.^86^ Confounding factors, such as comorbid psychiatric conditions and varying degrees of substance use severity, are not uniformly controlled. Additionally, variability in imaging methods, statistical thresholds, and heterogeneity in experimental paradigms and types of cues used make direct comparisons across studies difficult.^87^

This heterogeneity strengthens rather than weakens the conclusions of our meta-analysis. Across diverse substances, treatment types, and imaging designs, cue-elicited activation consistently localized to the cingulate gyrus, middle frontal gyrus, caudate, and insula. The current analysis defined regions associated with substance use and cessation across clinical^88^ and preclinical^89^ research using complementary methods, such as lesion mapping and pharmacologic manipulations, underscoring the potential of these regions as reliable biomarkers and advancing their applications in personalized SUD treatment strategies. The convergence of predictive biomarker and response biomarker findings in the insula in particular underscores its central role in both relapse risk and treatment-related change.

## Conclusions

In this meta-analysis of fMRI cue reactivity studies in adults undergoing treatment for SUDs, fMRI-based cue-reactivity measures appear promising and have potential as biomarkers, but the current evidence base is not yet sufficient to support their use as established tools for clinical decision-making. Their most appropriate role may be as exploratory end points or as mediating and moderating variables in future trials. Because none of these biomarkers have received US Food and Drug Administration approval at this time, a critical next step is to take these biomarker regions and validate them in randomized clinical trials.^90^ Future studies should prioritize harmonized methods and diverse samples to validate and extend these findings, focusing on not only changes in brain activation but also how these changes interact with behavioral outcomes in clinical settings. Future studies that integrate standardized effect size reporting, larger and more representative samples, and tests of causal or mediating roles will be critical for determining whether these neural measures can be translated into clinically actionable biomarkers.

## Notes

Supplement 1. **eMethods.** Supplementary methods **eTable 1.** Description of study demographics **eTable 2.** Description of study characteristics **eTable 3.** Omnibus meta-analysis results **eTable 4.** Predictive biomarker meta-analysis results **eTable 5.** Response biomarker (time) meta-analysis results **eTable 6.** Response biomarker (time x treatment) meta-analysis results **eTable 7.** Omnibus meta-analysis results: alcohol subgroup **eTable 8.** Omnibus meta-analysis results: pharmacological treatment subgroup **eTable 9.** Omnibus meta-analysis results: psychosocial treatment subgroup **eFigure 1.** Brain activation to drug cues over neutral in omnibus meta-analysis **eFigure 2.** Cue reactivity as a predictive biomarker **eFigure 3.** Cue reactivity as a response biomarker (time) **eFigure 4.** Cue reactivity as a response biomarker (time x treatment) **eFigure 5.** Funnel plots for significant clusters identified in the SDM meta-analysis

Supplement 2.
Data Sharing Statement
