# Supplementary material for: Cue-Elicited Brain Activity and Treatment Outcomes in Substance Use Disorders: A Meta-Analysis
Source: JAMA Netw Open. 2025 Dec 11;8(12):e2548809. doi: 10.1001/jamanetworkopen.2025.48809 (PMC12699361; doi:10.1001/jamanetworkopen.2025.48809)
Supplement: Supplement 1. — eMethods. Supplementary methods eTable 1. Description of study demographics eTable 2. Description of study characteristics eTable 3. Omnibus meta-analysis results eTable 4. Predictive biomarker meta-analysis results eTable 5. Response biomarker (time) meta-analysis results eTable 6. Response biomarker (time x treatment) meta-analysis results eTable 7. Omnibus meta-analysis results: alcohol subgroup eTable 8. Omnibus meta-analysis results: pharmacological treatment subgroup eTable 9. Omnibus meta-analysis results: psychosocial treatment subgroup eFigure 1. Brain activation to drug cues over neutral in omnibus meta-analysis eFigure 2. Cue reactivity as a predictive biomarker eFigure 3. Cue reactivity as a response biomarker (time) eFigure 4. Cue reactivity as a response biomarker (time x treatment) eFigure 5. Funnel plots for significant clusters identified in the SDM meta-analysis [file jamanetwopen-e2548809-s001.pdf]

Supplemental Online Content

Evohr B, Wenzzel S, Chen N, et al. Cue-elicited brain activity and treatment outcomes in substance use disorders: a meta-analysis. *JAMA Netw Open*. 2025;8(12):e2548809. doi:10.1001/jamanetworkopen.2025.48809

eMethods
eTable 1. Description of study demographics
eTable 2. Description of study characteristics
eTable 3. Omnibus meta-analysis results
eTable 4. Predictive biomarker meta-analysis results
eTable 5. Response biomarker (time) meta-analysis results
eTable 6. Response biomarker (time x treatment) meta-analysis results
eTable 7. Omnibus meta-analysis results: alcohol subgroup
eTable 8. Omnibus meta-analysis results: pharmacological treatment subgroup
eTable 9. Omnibus meta-analysis results: psychosocial treatment subgroup
eFigure 1. Brain activation to drug cues over neutral in omnibus meta-analysis
eFigure 2. Cue reactivity as a predictive biomarker
eFigure 3. Cue reactivity as a response biomarker (time)
eFigure 4. Cue reactivity as a response biomarker (time x treatment)
eFigure 5. Funnel plots for significant clusters identified in the SDM meta-analysis

© 2025 Evohr B et al. *JAMA Network Open*.

eMethods

Search Strategy

Database (Platform)	Search
PubMed (National Library of Medicine)	1. (((("Substance-Related Disorders"[Mesh] OR "Alcohol Drinking"[Mesh] OR "Drug-Seeking Behavior"[Mesh] OR "tobacco use"[Mesh] OR "nicotine"[Mesh] OR "Smoking"[Mesh]) AND ("drug therapy"[MeSH Subheading] OR "rehabilitation"[MeSH Subheading] OR "therapy"[MeSH Subheading] OR "psychology"[Mesh Subheading])) OR "Naloxone"[Mesh] OR "Buprenorphine, Naloxone Drug Combination"[Mesh] OR "Buprenorphine"[Mesh] OR "Substance Abuse Treatment Centers"[Mesh] OR "Tobacco Use Cessation"[Mesh]) OR ((((substance*[tiab] OR drug[tiab] OR narcotic*[tiab] OR cocaine[tiab] OR crack[tiab] OR opium[tiab] OR opiate[tiab] OR opioid[tiab] OR oxycontin[tiab] OR oxycodone[tiab] OR hydrocodeine[tiab] OR Dihydrocodeine[tiab] OR hydrocodone[tiab] OR heroin[tiab] OR fentanyl[tiab] OR dilaudid[tiab] OR hydromorphone[tiab] OR alcohol[tiab] OR tobacco[tiab] OR nicotine[tiab] OR smoking[tiab] OR drinking[tiab] OR cannabis[tiab] OR weed[tiab] OR marijuana[tiab] OR THC[tiab] OR speed[tiab] OR amphetamine*[tiab] OR meth[tiab] OR methamphetamine*[tiab] OR polysubstance[tiab] OR drinker*[tiab]) AND (use[tiab] OR misuse[tiab] OR abuse[tiab] OR addict*[tiab] OR dependen*[tiab] OR habituat*[tiab] OR withdrawal[tiab] OR overdose*[tiab] OR problem[tiab])) OR smoker*[tiab] OR alcoholic*[tiab]) AND (treatment[tiab] OR outpatient[tiab] OR rehab*[tiab] OR "out patient"[tiab] OR inpatient[tiab] OR "in patient"[tiab] OR therap*[tiab] OR diversion[tiab] OR cessation[tiab] OR methadone[tiab] OR suboxone[tiab] OR buprenorphine[tiab] OR naloxone[tiab] OR naltrexone[tiab]))) AND ("Cues"[Mesh] OR (cue[tiab] OR cues[tiab]) AND (activ*[tiab] OR reactiv*[tiab] OR smok*[tiab] OR tobacco[tiab] OR cigarette[tiab] OR alcohol[tiab] OR drinking[tiab] OR drug*[tiab] OR nicotine[tiab] OR opiate[tiab] OR heroin[tiab] OR crav*[tiab] OR narcotic[tiab] OR stimul*[tiab] OR respons*[tiab])) AND ("Brain/diagnostic imaging"[Mesh] OR "Brain Mapping"[Mesh] OR "Magnetic Resonance Imaging"[Mesh:Noexp] OR MRI[tiab] OR "functional magnetic resonance"[tiab] OR fMRI[tiab])<br>2. ("Animals"[Mesh] OR "Animal Experimentation"[Mesh] OR "Models, Animal"[Mesh] OR (rat OR rats OR mouse OR mice OR rodent* OR swine OR porcine OR piglet* OR pig OR murine OR sheep OR lamb OR lambs OR rabbit* OR hare OR cat OR cats OR feline OR canine* or dog OR dogs OR bovine OR cattle OR cow OR cows OR monkey OR marmoset* OR trout OR zebrafish OR fish OR frog OR amphibian* OR finch OR bird OR birds OR hamster* OR gerbil* OR chinchilla* OR armadillo* OR rhesus OR macaque*)) NOT "Humans"[Mesh]<br>3. #1 NOT #2
Embase (Elsevier)	1. (('substance use'/exp OR 'recreational drug use'/exp OR 'cocaine'/exp OR 'cannabis'/exp OR 'methamphetamine'/exp OR 'opiate'/exp OR 'hydrocodone'/exp OR 'hydrocodone'/exp OR 'oxycodone'/exp OR 'dihydrocodeine'/exp OR 'tobacco'/exp OR 'hydromorphone'/exp OR 'diamorphine'/exp OR 'fentanyl'/exp OR 'drug dependence'/exp OR 'drug abuse'/exp) AND ('rehabilitation'/exp OR 'drug therapy'/exp OR 'counseling'/exp OR 'psychology'/exp)) OR ('drugs used in the treatment of addiction'/exp OR 'withdrawal syndrome'/exp OR 'smoking cessation'/exp OR 'naloxone'/exp OR 'alcohol rehabilitation'/de OR 'alcoholics anonymous'/de OR 'community based rehabilitation'/de OR 'drug dependence treatment'/exp OR 'narcotics anonymous'/exp) OR ((((substance* OR drug OR narcotic* OR cocaine OR crack OR opium OR opiate OR opioid OR oxycontin OR oxycodone OR hydrocodeine OR Dihydrocodeine OR hydrocodone OR heroin

© 2025 Evohr B et al. *JAMA Network Open*.

	OR fentanyl OR dilaudid OR hydromorphone OR alcohol OR tobacco OR nicotine OR smoking OR drinking OR cannabis OR weed OR marijuana OR THC OR speed OR amphetamine* OR meth OR methamphetamine* OR polysubstance OR drinker* OR polydrug*) NEAR/3 (use OR user OR misuse OR abuse OR addict* OR dependen* OR habituat* OR withdrawal OR overdose* OR problem)) OR smoker* OR alcoholic*) AND (treatment OR outpatient OR rehab* OR 'out patient' OR inpatient OR 'in patient' OR therap* OR diversion OR cessation OR methadone OR suboxone OR buprenorphine OR naloxone OR naltrexone)):ti,ab,kw<br>2. 'association'/mj/exp OR 'drug craving'/exp OR 'craving'/de OR ((cue OR cues OR cued) AND (activ* OR reactiv* OR smok* OR tobacco OR cigarette* OR alcohol OR drinking OR drug* OR nicotine OR opiate OR heroin OR crav* OR narcotic OR stimul* OR respons*)):ti,ab,kw<br>3. 'functional magnetic resonance imaging'/exp OR 'brain mapping'/exp OR 'nuclear magnetic resonance imaging'/de OR (MRI OR "functional magnetic resonance" OR fMRI):ti,ab,kw<br>4. #1 AND #2 AND #3 AND [embase]/lim<br>5. 'Animal'/exp OR 'Veterinary Study'/exp OR 'Human Versus Animal Comparison'/exp OR 'nonhuman'/exp OR (rat OR rats OR mouse OR mice OR rodent* OR swine OR porcine OR piglet* OR murine OR sheep OR lambs OR pig OR pigs OR rabbit* OR cat OR cats OR feline* OR canine* OR dog OR dogs OR cattle OR bovine OR ovine OR monkey* OR marmoset* OR trout OR zebrafish OR fish OR frog OR amphibian* OR finch OR bird OR birds OR hamster* OR gerbil* OR chinchilla* OR armadillo* OR rhesus OR macaque*)<br>6. 'Human'/exp<br>7. #4 NOT (#5 NOT #6)
APA PsycInfo (EBSCO)	1. DE (("Drug Abuse" OR "Polydrug Abuse" OR "Drug Addiction" OR OR "Drug Dependency" OR "Substance Use Disorder" OR "Alcoholism" OR "Alcohol Induced Psychotic Disorders" OR "Alcohol Use Disorder" OR "Alcohol Abuse" OR "Alcohol Intoxication" OR "Substance Related and Addictive Disorders" OR "Addiction" OR "Nicotine" OR OR "Methadone" OR "Opiates" OR "Codeine" OR "Endogenous Opiates" OR "Heroin" OR "Morphine" OR OR "Prescription Drug Misuse" OR "Cannabis Use Disorder" OR "Inhalant Abuse" OR "Glue Sniffing" OR "Opioid Use Disorder" OR "Heroin Use Disorder" OR "Morphine Dependence" OR "Tobacco Use Disorder" OR "Cannabis" OR "Cannabinoids" OR "Hashish" OR "Marijuana" OR "Cocaine" OR "Crack Cocaine" OR "Methamphetamine" OR "Buprenorphine" OR "Fentanyl" OR "Oxycodone" OR "Drug Overdoses" OR "Cannabis Use") AND ("Addiction Treatment" OR "Detoxification" OR "Medication-Assisted Treatment" OR "Methadone Maintenance" OR "Twelve Step Programs" OR "Treatment" OR "Alcoholics Anonymous" OR "Alcohol Withdrawal" OR "Substance Use Treatment" "Drug Withdrawal" OR "Alcohol Treatment" OR "Nicotine Withdrawal" OR "Smoking Cessation" OR "Opioid Withdrawal")) OR TI ((((substance* OR drug OR narcotic* OR cocaine OR crack OR opium OR opiate OR opioid OR oxycontin OR oxycodone OR hydrocodeine OR Dihydrocodeine OR hydrocodone OR heroin OR fentanyl OR dilaudid OR hydromorphone OR alcohol OR tobacco OR nicotine OR smoking OR drinking OR cannabis OR weed OR marijuana OR THC OR speed OR amphetamine* OR meth OR methamphetamine* OR polysubstance OR drinker* OR polydrug*) N3 (use OR user OR misuse OR abuse OR addict* OR dependen* OR habituat* OR withdrawal OR overdose* OR problem)) OR smoker* OR alcoholic*) AND (treatment OR outpatient OR rehab* OR "out patient" OR inpatient OR "in patient" OR therap* OR diversion OR cessation OR methadone OR suboxone OR buprenorphine OR naloxone OR naltrexone)) OR AB ((((substance* OR drug OR narcotic* OR cocaine OR crack OR opium OR opiate OR opioid OR oxycontin OR oxycodone OR hydrocodeine OR Dihydrocodeine OR hydrocodone OR heroin OR fentanyl OR dilaudid

© 2025 Evohr B et al. *JAMA Network Open*.

	OR hydromorphone OR alcohol OR tobacco OR nicotine OR smoking OR drinking OR cannabis OR weed OR marijuana OR THC OR speed OR amphetamine* OR meth OR methamphetamine* OR polysubstance OR drinker* OR polydrug*) N3 (use OR user OR misuse OR abuse OR addict* OR dependen* OR habituat* OR withdrawal OR overdose* OR problem)) OR smoker* OR alcoholic*) AND (treatment OR outpatient OR rehab* OR "out patient" OR inpatient OR "in patient" OR therap* OR diversion OR cessation OR methadone OR suboxone OR buprenorphine OR naloxone OR naltrexone))<br>2. DE ("Craving" OR "Cues") OR TI ((cue OR cues OR cued) AND (activ* OR reactiv* OR smok* OR tobacco OR cigarette* OR alcohol OR drinking OR drug* OR nicotine OR opiate OR heroin OR crav* OR narcotic OR stimul* OR respons*)) OR AB ((cue OR cues OR cued) AND (activ* OR reactiv* OR smok* OR tobacco OR cigarette* OR alcohol OR drinking OR drug* OR nicotine OR opiate OR heroin OR crav* OR narcotic OR stimul* OR respons*))<br>3. DE ("Substance Abuse and Addiction Measures" OR "Magnetic Resonance Imaging" OR "Functional Magnetic Resonance Imaging") OR TI (MRI OR "functional magnetic resonance" OR fMRI) OR AB (MRI OR "functional magnetic resonance" OR fMRI)<br>4. S1 AND S2 AND S3
Web of Science (Clarivate) [Indices searched: SCI-EXP, SSCI, AHCI, CPCI-S, CPCI-SSH, BKCI-S, BKCI-SSH, ESCI, CCR-EXP, IC]	1. TI=((((substance* OR drug OR narcotic* OR cocaine OR crack OR opium OR opiate OR opioid OR oxycontin OR oxycodone OR hydrocodeine OR Dihydrocodeine OR hydrocodone OR heroin OR fentanyl OR dilaudid OR hydromorphone OR alcohol OR tobacco OR nicotine OR smoking OR drinking OR cannabis OR weed OR marijuana OR THC OR speed OR amphetamine* OR meth OR methamphetamine* OR polysubstance OR drinker* OR polydrug*) NEAR/3 (use OR user OR misuse OR abuse OR addict* OR dependen* OR habituat* OR withdrawal OR overdose* OR problem)) OR smoker* OR alcoholic*) AND (treatment OR outpatient OR rehab* OR "out patient" OR inpatient OR "in patient" OR therap* OR diversion OR cessation OR methadone OR suboxone OR buprenorphine OR naloxone OR naltrexone)) OR AB=((((substance* OR drug OR narcotic* OR cocaine OR crack OR opium OR opiate OR opioid OR oxycontin OR oxycodone OR hydrocodeine OR Dihydrocodeine OR hydrocodone OR heroin OR fentanyl OR dilaudid OR hydromorphone OR alcohol OR tobacco OR nicotine OR smoking OR drinking OR cannabis OR weed OR marijuana OR THC OR speed OR amphetamine* OR meth OR methamphetamine* OR polysubstance OR drinker* OR polydrug*) NEAR/3 (use OR user OR misuse OR abuse OR addict* OR dependen* OR habituat* OR withdrawal OR overdose* OR problem)) OR smoker* OR alcoholic*) AND (treatment OR outpatient OR rehab* OR "out patient" OR inpatient OR "in patient" OR therap* OR diversion OR cessation OR methadone OR suboxone OR buprenorphine OR naloxone OR naltrexone)) OR AK=((((substance* OR drug OR narcotic* OR cocaine OR crack OR opium OR opiate OR opioid OR oxycontin OR oxycodone OR hydrocodeine OR Dihydrocodeine OR hydrocodone OR heroin OR fentanyl OR dilaudid OR hydromorphone OR alcohol OR tobacco OR nicotine OR smoking OR drinking OR cannabis OR weed OR marijuana OR THC OR speed OR amphetamine* OR meth OR methamphetamine* OR polysubstance OR drinker* OR polydrug*) NEAR/3 (use OR user OR misuse OR abuse OR addict* OR dependen* OR habituat* OR withdrawal OR overdose* OR problem)) OR smoker* OR alcoholic*) AND (treatment OR outpatient OR rehab* OR "out patient" OR inpatient OR "in patient" OR therap* OR diversion OR cessation OR methadone OR suboxone OR buprenorphine OR naloxone OR naltrexone))<br>2. TI=((cue OR cues OR cued) AND (activ* OR reactiv* OR smok* OR tobacco OR cigarette* OR alcohol OR drinking OR drug* OR nicotine OR opiate OR heroin OR crav* OR narcotic OR stimul* OR respons*)) OR AB=((cue OR cues OR cued) AND (activ* OR reactiv* OR smok* OR tobacco OR cigarette* OR alcohol OR drinking OR drug* OR nicotine OR

© 2025 Evohr B et al. *JAMA Network Open*.

	opiate OR heroin OR crav* OR narcotic OR stimul* OR respons*)) OR AK=((cue OR cues OR cued) AND (activ* OR reactiv* OR smok* OR tobacco OR cigarette* OR alcohol OR drinking OR drug* OR nicotine OR opiate OR heroin OR crav* OR narcotic OR stimul* OR respons*))<br>3. TI=(MRI OR "functional magnetic resonance" OR fMRI) OR AB=(MRI OR "functional magnetic resonance" OR fMRI) OR AK=(MRI OR "functional magnetic resonance" OR fMRI)<br>4. #1 AND #2 AND #3
Cochrane Library (Wiley)	1. (substance* OR drug OR narcotic* OR cocaine OR crack OR opium OR opiate OR opioid OR oxycontin OR oxycodone OR hydrocodeine OR Dihydrocodeine OR hydrocodone OR heroin OR fentanyl OR dilaudid OR hydromorphone OR alcohol OR tobacco OR nicotine OR smoking OR drinking OR cannabis OR weed OR marijuana OR THC OR speed OR amphetamine* OR meth OR methamphetamine* OR polysubstance OR drinker* OR polydrug*):ti,ab,kw<br>2. (use OR user OR misuse OR abuse OR addict* OR dependen* OR habituat* OR withdrawal OR overdose* OR problem):ti,ab,kw<br>3. (smoker* OR alcoholic*):ti,ab,kw<br>4. (treatment OR outpatient OR rehab* OR "out patient" OR inpatient OR "in patient" OR therap* OR diversion OR cessation OR methadone OR suboxone OR buprenorphine OR naloxone OR naltrexone):ti,ab,kw<br>5. ((1 AND 2) OR 3) AND 4<br>6. (cue OR cues OR cued):ti,ab,kw<br>7. (activ* OR reactiv* OR smok* OR tobacco OR cigarette* OR alcohol OR drinking OR drug* OR nicotine OR opiate OR heroin OR crav* OR narcotic OR stimul* OR respons*):ti,ab,kw<br>8. 6 AND 7<br>9. (MRI OR "functional magnetic resonance" OR fMRI):ti,ab,kiw<br>10. 5 AND 8 AND 9

© 2025 Evohr B et al. *JAMA Network Open*.

eTable 1: Description of study demographics

Study	Primary Substance	N	% Female	Age	Abstinent/ Detoxified Before Treatment
Allenby 2020	Nicotine	75	46.7	43 (12.7)	No
Bach 2020	Alcohol	90	0	44.2 (9.3)	Yes
Beck 2018	Alcohol	23	30.4	46.2 (6.3)	Yes
Blaine 2020	Alcohol	87	41.4	34.1 (10.7)	Yes
Bradstreet 2014	Nicotine	30	36.7	25.7 (9.4)	No
Burnette 2021	Alcohol	45	35.6	32.5 (8.5)	No
Culbertson 2011	Nicotine	30	30	41.7 (3)	No
Elkins 2017	Alcohol	13	23.1	45 (6.83)	Yes
Franklin 2011	Nicotine	22	27.3	36.1 (2.2)	No
Froeliger 2017	Nicotine	13	30.8	49 (12.7)	No
Grieder 2022	Alcohol	45	35.6	43.4 (9.5)	Yes
Grodin 2021	Alcohol	45	40	32.6 (8.5)	No
Grodin 2022	Alcohol	51	33.3	32.7 (8.1)	No
Han 2013	Alcohol	35	34.3	39.6 (7.7)	Yes
Hartwell 2013	Nicotine	21	57.1	35.2 (12.1)	No
Herremans 2015	Alcohol	26	34.6	45.2 (9.3)	Yes
Herremans 2016	Alcohol	19	42.1	44.9 (6.4)	Yes
Holla 2018	Alcohol	35	0	36.2 (7.2)	Yes

© 2025 Evohr B et al. *JAMA Network Open*.

Study	Primary Substance	N	% Female	Age	Abstinent/ Detoxified Before Treatment
Janes 2009	Nicotine	13	100	43.2 (11.5)	No
Janes 2010	Nicotine	21	100	Not Reported	No
Karch 2019	Nicotine	22	50	43.83 (12.37)	No
Karch 2022	Alcohol	48	14.6	45.1 (13.3)	Yes
Ketcherside 2020	Nicotine	43	37.2	39.6 (12)	No
Kiefer 2015	Alcohol	56	23.2	45.8 (10.3)	Yes
Klausen 2022	Alcohol	56	91.1	52.3 (10.4)	No
Kosten 2006	Stimulants	17	29.4	37 (4)	Yes
Langleben 2008	Opioids	15	80	36 (11)	Yes
Langleben 2014	Opioids	13	15.4	36 (8.4)	Yes
Li 2015	Opioids	44	0	35.1 (7)	Yes
Li 2020	Nicotine	24	41.7	26.08 (4.53)	No
Logge 2021	Alcohol	22	45.5	50.1 (10.8)	Yes
Lukas 2013	Alcohol	28	32.1	46.6 (9.2)	Yes
MacNiven 2018	Stimulants	76	23.7	37.4 (12.4)	Yes
Machielsen 2014	Cannabis	47	0	22.4 (3.1)	Yes
Machielsen 2018	Cannabis	50	0	22.4 (3)	Yes
Mann 2014	Alcohol	73	17.8	43.2 (8.5)	Yes
Matto 2014	Polysubstance	10	70	40.7	Yes

© 2025 Evohr B et al. *JAMA Network Open*.

Study	Primary Substance	N	% Female	Age	Abstinent/ Detoxified Before Treatment
Mayer 2020	Stimulants	37	37.8	38.3 (10.9)	No
Mondino 2018	Nicotine	24	83.3	41 (9.2)	No
Owens 2018	Nicotine	32	43.8	39.1 (11.9)	No
Prisciandaro 2013a	Stimulants	30	83.3	47 (8.6)	Yes
Prisciandaro 2013b	Stimulants	25	8	45.7 (10.7)	Yes
Schacht 2013	Alcohol	48	22.9	46.8 (10.7)	No
Schneider 2001	Alcohol	20	0	41.4 (7.7)	Yes
Schulte 2019	Stimulants	24	0	37 (8.1)	No
Shi 2018	Opioids	24	37.5	30.21 (8.47)	Yes
Vollstadt-Klein 2011	Alcohol	30	36.7	46.5 (8.5)	Yes
Wang 2015	Opioids	32	46.9	29.19 (7.5)	Yes
Wetherill 2021	Alcohol	20	30	48.3 (10.4)	No
Wiers 2015a	Alcohol	26	0	45.23 (7.03)	Yes
Wiers 2015b	Alcohol	32	0	45.33 (6.84)	Yes

© 2025 Evohr B et al. *JAMA Network Open*.

eTable 2: Description of study characteristics

Study	Included Meta-Analyses	Treatment	Study Design	Cue Type	Time Between Scans	Predictive Biomarker Outcome
Allenby 2020	Omnibus	Individual counseling sessions	Non-randomized clinical trial	Visual (Images)	1-2 weeks	
Bach 2020	Omnibus, Response biomarker (time, time x treatment)	Naltrexone, intensive withdrawal treatment	Non-randomized clinical trial	Visual (Images)	1-3 months	
Beck 2018	Omnibus, Response biomarker (time, time x treatment)	Baclofen	Randomized controlled trial	Visual (Images)	1-3 months	
Blaine 2020	Omnibus, Predictive biomarker	12-step NIAAA weekly behavioral counseling	Non-randomized clinical trial	Visual (Images)	Baseline only	Direct: Abstinence days, Heavy drinking days
Bradstreet 2014	Omnibus	Contingency management	Randomized controlled trial	Visual (Images)	1-2 weeks	
Burnette 2021	Omnibus	Ibudilast	Randomized controlled trial	Visual (Images)	Baseline only	
Culbertson 2011	Omnibus, Response biomarker (time, time x treatment)	Bupropion	Randomized controlled trial	Audio-Visual	1-3 months	
Elkins 2017	Omnibus, Response biomarker (time)	Chemical aversion conditioning (Ipecac)	Non-randomized clinical trial	Visual (Images)	1-2 weeks	

© 2025 Evohr B et al. *JAMA Network Open*.

Study	Included Meta-Analyses	Treatment	Study Design	Cue Type	Time Between Scans	Predictive Biomarker Outcome
Franklin 2011	Omnibus, Predictive biomarker	Varenicline	Randomized controlled trial	Audio-Visual	3-4 weeks	Proxy: Craving
Froeliger 2017	Omnibus, Response biomarker (time x treatment)	Mindfulness-Oriented Recovery Enhancement (MORE)	Randomized controlled trial	Visual (Images)	1-3 months	
Grieder 2022	Omnibus, Predictive biomarker	Alcohol-specific inhibition training	Randomized controlled trial	Visual (Images)	1-3 months	Direct: Change in % days abstinent
Grodin 2021	Omnibus	Ibudilast	Randomized controlled trial	Visual (Images)	Baseline only	
Grodin 2022	Omnibus	Ibudilast	Randomized controlled trial	Visual (Images)	Baseline only	
Han 2013	Omnibus	Escitalopram augmented with aripiprazole	Randomized controlled trial	Visual (Images)	1-3 months	
Hartwell 2013	Omnibus, Predictive biomarker, Response biomarker (time)	Varenicline	Non-randomized clinical trial	Visual (Images)	1-3 months	Direct: Abstinence
Herremans 2015	Omnibus, Response biomarker (time, time x treatment)	Transcranial magnetic stimulation	Non-randomized clinical trial	Visual (Images)	< 1 week	
Herremans 2016	Omnibus, Predictive biomarker, Response biomarker (time)	Transcranial magnetic stimulation	Non-randomized clinical trial	Visual (Images)	< 1 week	Direct: Abstinence

© 2025 Evohr B et al. *JAMA Network Open*.

Study	Included Meta-Analyses	Treatment	Study Design	Cue Type	Time Between Scans	Predictive Biomarker Outcome
Holla 2018	Omnibus	Baclofen	Non-randomized clinical trial	Visual (Images)	1-2 weeks	
Janes 2009	Omnibus, Response biomarker (time)	Nicotine replacement therapy	Non-randomized clinical trial	Visual (Images)	1-3 months	
Janes 2010	Omnibus, Predictive biomarker	Nicotine replacement therapy and weekly manualized individual behavioral intervention	Non-randomized clinical trial	Visual (Images)	Baseline only	Direct: Abstinence
Karch 2019	Omnibus, Predictive biomarker	Neurofeedback, group-based psychotherapy	Randomized controlled trial	Visual (Images)	< 1 week	Direct: Abstinence
Karch 2022	Omnibus, Predictive biomarker, Response biomarker (time)	Neurofeedback	Randomized controlled trial	Visual (Images)	1-2 weeks	Direct: Abstinence
Ketcherside 2020	Omnibus, Response biomarker (time x treatment)	Baclofen	Randomized controlled trial	Audio-Visual	3-4 weeks	
Kiefer 2015	Omnibus, Response biomarker (time)	D-cycloserine before cue-exposure-based extinction training	Randomized controlled trial	Imagery, Olfaction	3-4 weeks	
Klausen 2022	Omnibus, Response biomarker (time)	Exenatide, cognitive behavioral therapy	Randomized controlled trial	Visual (Images)	> 3 months	

© 2025 Evohr B et al. *JAMA Network Open*.

Study	Included Meta-Analyses	Treatment	Study Design	Cue Type	Time Between Scans	Predictive Biomarker Outcome
Kosten 2006	Omnibus, Predictive biomarker	Sertraline, cognitive behavioral therapy	Randomized controlled trial	Audio-Visual	Baseline only	Direct: Cocaine-free urine drug tests, Abstinence
Langleben 2008	Omnibus, Response biomarker (time)	Methadone maintenance treatment	Cohort study	Visual (Images)	3-4 weeks	
Langleben 2014	Omnibus, Response biomarker (time)	Naltrexone	Cohort study	Visual (Images)	1-2 weeks	
Li 2015	Omnibus	Methadone maintenance treatment	Cohort study	Visual (Images)	Baseline only	
Li 2020	Omnibus	Hypnotherapy	Non-randomized clinical trial	Visual (Images)	< 1 week	
Logge 2021	Omnibus	Baclofen	Randomized controlled trial	Visual (Images)	Baseline only	
Lukas 2013	Omnibus	Naltrexone, weekly counseling sessions	Randomized controlled trial	Olfaction	1-2 weeks	
MacNiven 2018	Omnibus	28-day residential treatment program	Cohort study	Visual (Images)	Baseline only	
Machielsen 2014	Omnibus, Predictive biomarker, Response biomarker (time, time x treatment)	Clozapine or risperidone	Randomized controlled trial	Imagery	3-4 weeks	Proxy: Craving

© 2025 Evohr B et al. *JAMA Network Open*.

Study	Included Meta-Analyses	Treatment	Study Design	Cue Type	Time Between Scans	Predictive Biomarker Outcome
Machielsen 2018	Omnibus, Predictive biomarker, Response biomarker (time x treatment)	Clozapine or risperidone	Randomized controlled trial	Visual (Images)	3-4 weeks	Proxy: Craving
Mann 2014	Omnibus	Naltrexone, acamprosate	Randomized controlled trial	Visual (Images)	Baseline only	
Matto 2014	Omnibus, Response biomarker (time)	Dual-processing relapse intervention	Randomized controlled trial	Visual (Images)	1-3 months	
Mayer 2020	Omnibus	Attentional bias modification therapy	Randomized controlled trial	Visual (Videos)	3-4 weeks	
Mondino 2018	Omnibus, Response biomarker (time x treatment)	Transcranial direct current stimulation	Randomized controlled trial	Visual (Images)	< 1 week	
Owens 2018	Omnibus, Predictive biomarker	Nicotine replacement therapy, individual counseling sessions	Non-randomized clinical trial	Visual (Images)	Baseline only	Direct: Longer days to lapse
Prisciandaro 2013a	Omnibus, Predictive biomarker	D-cycloserine before cue extinction sessions	Randomized controlled trial	Visual (Images)	Baseline only	Direct: Positive urine drug test
Prisciandaro 2013b	Omnibus, Response biomarker (time)	D-cycloserine before cue extinction sessions	Randomized controlled trial	Visual (Images)	1-2 weeks	

© 2025 Evohr B et al. *JAMA Network Open*.

Study	Included Meta-Analyses	Treatment	Study Design	Cue Type	Time Between Scans	Predictive Biomarker Outcome
Schacht 2013	Omnibus	Gabapentin, flumazenil	Randomized controlled trial	Visual (Images)	Baseline only	
Schneider 2001	Omnibus	Cognitive behavioral therapy	Non-randomized clinical trial	Olfaction	3-4 weeks	
Schulte 2019	Omnibus	N-acetylcysteine, working memory training	Randomized controlled trial	Visual (Images)	3-4 weeks	
Shi 2018	Omnibus, Response biomarker (time)	Naltrexone	Non-randomized clinical trial	Visual (Images)	1-2 weeks	
Vollstadt-Klein 2011	Omnibus, Response biomarker (time x treatment)	Cue-exposure based extinction training	Randomized controlled trial	Visual (Images)	3-4 weeks	
Wang 2015	Omnibus, Predictive biomarker	Naltrexone	Non-randomized clinical trial	Visual (Images)	> 3 months	Proxy: Number of naltrexone injections (treatment retention)
Wetherill 2021	Omnibus, Response biomarker (time x treatment)	Topiramate	Randomized controlled trial	Audio-Visual	1-3 months	
Wiers 2015a	Omnibus, Response biomarker (time, time x treatment)	Cognitive bias modification	Randomized controlled trial	Visual (Images)	3-4 weeks	
Wiers 2015b	Omnibus, Response biomarker (time, time x treatment)	Cognitive bias modification	Randomized controlled trial	Visual (Images)	3-4 weeks	

© 2025 Evohr B et al. *JAMA Network Open*.

eTable 3: Omnibus meta-analysis results

Region	MNI Coordinate	Uncorrected			TFCE Corrected			Heterogeneity I^2	Metabias P-Value	Excess Significance P-Value
		Voxels	Z Score	P-Value	Voxels	Z Score	P-Value			
Left anterior cingulate / paracingulate gyri, BA 10	-8, 54, 2	1477	5.833	< 0.001	865	5.833	< 0.001	14.706	0.693	0.874
Bilateral posterior cingulate	0, -50, 18	1107	5.864	< 0.001	443	5.864	0.002	16.31	0.852	< 0.001
Right insula, BA 48	44, 8, 0	800	4.83	< 0.001	84	4.83	0.021	6.875	0.292	1
Left inferior temporal gyrus, BA 37	-46, -68, -8	563	5.143	< 0.001				24.886	0.037	1
Right calcarine fissure / surrounding cortex, BA 18	18, -92, 4	401	4.789	< 0.001				9.005	0.396	0.624
Right inferior temporal gyrus, BA 19	46, -74, -6	342	4.052	< 0.001				57.75	0.1	0.999
Right inferior network, inferior longitudinal fasciculus	44, 4, -30	282	3.886	< 0.001				0.163	0.948	0.936
Bilateral thalamus	4, -22, -6	238	4.835	< 0.001				14.066	0.434	0.201
Left median cingulate / paracingulate gyri, BA 32	-6, 28, 32	217	3.722	< 0.001				6.35	0.953	1
Right superior parietal gyrus, BA 7	26, -60, 50	160	3.809	< 0.001				26.83	0.186	0.999
Left superior temporal gyrus, BA 48	-56, -6, 6	161	3.496	< 0.001				4.919	0.229	0.624
Right anterior thalamic projections	16, 4, 18	120	4.55	< 0.001				1.311	0.933	0.271

© 2025 Evohr B et al. *JAMA Network Open*.

Region	MNI Coordinate	Uncorrected			TFCE Corrected			Heterogeneity I^2	Metabias P-Value	Excess Significance P-Value
		Voxels	Z Score	P-Value	Voxels	Z Score	P-Value			
Left angular gyrus, BA 7	-36, -62, 44	132	3.369	< 0.001				25.874	0.487	1
Right angular gyrus, BA 22	54, -58, 24	72	3.594	< 0.001				44.537	0.866	0.977
Left hippocampus, BA 20	-28, -16, -18	24	3.215	< 0.001				2.418	0.637	0.998
Left middle frontal gyrus, BA 46	-34, 30, 38	22	3.495	< 0.001				2.032	0.575	< 0.001

eTable 4: Predictive biomarker meta-analysis results

Region	MNI Coordinate	Uncorrected			TFCE Corrected			Heterogeneity I^2	Metabias P-Value	Excess Significance P-Value
		Voxels	Z Score	P-Value	Voxels	Z Score	P-Value			
Right insula	38, -10, 0	118	-3.469	< 0.001				15.955	0.491	0.909
Right median cingulate / paracingulate gyri	6, -44, 38	34	-3.185	< 0.001				5.486	0.582	< 0.001
Left insula, BA 48	-30, 26, 6	29	-3.559	< 0.001				16.628	0.694	0.986
Left precuneus, BA 7	-2, -64, 38	27	-3.007	0.001				4.441	0.514	< 0.001
Left median cingulate / paracingulate gyri, BA 23	-2, -18, 42	16	-2.771	0.003				3.093	0.526	< 0.001
Subgenual anterior cingulate	0, 16, -16	16	3.035	0.001				2.401	0.793	0.984

eTable 5: Response biomarker (time) meta-analysis results

© 2025 Evohr B et al. *JAMA Network Open*.

Region	MNI Coordinate	Uncorrected			TFCE Corrected			Heterogeneity I^2	Metabias P-Value	Excess Significance P-Value
		Voxels	Z Score	P-Value	Voxels	Z Score	P-Value			
Right olfactory cortex, BA 25	4, 16, -6	211	-4.283	< 0.001				17.468	0.718	1
Left amygdala, BA 28	-24, -4, -24	197	-4.335	< 0.001	12	-4.335	0.033	0.971	0.752	0.741
Left insula, BA 48	-36, 18, 6	90	-3.308	< 0.001				26.232	0.827	0.242
Left parahippocampal gyrus, BA 28	-14, -12, -24	65	-3.686	< 0.001				11.817	0.797	0.793
Right lenticular nucleus, putamen, BA 48	30, 16, -2	64	-3.343	< 0.001				3.2	0.838	< 0.001

eTable 6: Response biomarker (time x treatment) meta-analysis results

Region	MNI Coordinate	Uncorrected			TFCE Corrected			Heterogeneity I^2	Metabias P-Value	Excess Significance P-Value
		Voxels	Z Score	P-Value	Voxels	Z Score	P-Value			
Right temporal pole, superior temporal gyrus, BA 38	38, 10, -26	454	-4.413	< 0.001	465	-4.413	< 0.001	0.622	0.885	0.976
Left striatum	-14, 8, -6	288	-3.389	< 0.001	96	-3.389	0.035	10.53	0.391	0.674
Right superior frontal gyrus, medial orbital, BA 11	2, 40, -14	203	-3.458	< 0.001	13	-3.458	0.047	9.436	0.345	0.292
Bilateral thalamus	0, -18, -6	46	-3.069	0.001				23.202	0.548	0.511
Right insula, BA 48	42, 8, -2	42	-3.302	< 0.001				1.468	0.808	0.292
Left insula, BA 48	-38, 14, -2	40	-3.294	< 0.001				1.125	0.806	0.901

© 2025 Evohr B et al. *JAMA Network Open*.

eTable 7: Omnibus meta-analysis results: alcohol subgroup

Region	MNI Coordinate	Uncorrected			TFCE Corrected			Heterogeneity I^2	Metabias P-Value	Excess Significance P-Value
		Voxels	Z Score	P-Value	Voxels	Z Score	P-Value			
Left superior frontal gyrus, medial, BA 10	-4, 58, 14	1102	5.291	< 0.001	338	5.291	< 0.001	16.176	0.895	0.997
Right insula, BA 48	40, 10, 2	890	4.242	< 0.001	154	4.242	0.029	0.058	0.982	0.999
Left supplementary motor area, BA 6	-4, 18, 64	425	4.643	< 0.001				16.858	0.976	1
Right caudate nucleus, BA 25	10, 18, 4	331	5.244	< 0.001				0.154	0.953	0.88
Right temporal pole, superior temporal gyrus	40, 12, -22	286	4.193	< 0.001				2.605	0.654	0.392
Right middle temporal gyrus, BA 39	56, -66, 20	215	3.737	< 0.001				11.148	0.79	0.994
Right inferior network, inferior fronto-occipital fasciculus	20, -92, 4	194	3.673	< 0.001				0.169	0.602	0.997
Left lenticular nucleus, putamen, BA 48	-30, -10, 8	190	3.946	< 0.001				0.075	0.635	0.373
Right inferior network, inferior fronto-occipital fasciculus	34, 30, -16	70	3.06	0.001				19.794	0.86	0.99
Left superior parietal gyrus, BA 7	-22, -62, 50	45	3.838	< 0.001				2.341	0.699	0.995
Right inferior temporal gyrus, BA 19	50, -74, -8	44	3.355	< 0.001				36.353	0.602	0.977

© 2025 Evohr B et al. *JAMA Network Open*.

Region	MNI Coordinate	Uncorrected			TFCE Corrected			Heterogeneity I^2	Metabias P-Value	Excess Significance P-Value
		Voxels	Z Score	P-Value	Voxels	Z Score	P-Value			
Bilateral posterior cingulate	0, -48, 18	30	2.983	0.001				25.424	0.905	1
Left anterior thalamic projections	-16, 18, 2	24	3.179	< 0.001				0.075	0.908	0.999
Left middle occipital gyrus, BA 19	-40, -84, -2	24	2.905	0.002				2.606	0.891	1
Left anterior thalamic projections	-4, -20, -2	13	3.107	< 0.001				8.862	0.849	0.988

eTable 8: Omnibus meta-analysis results: pharmacological treatment subgroup

Region	MNI Coordinate	Uncorrected			TFCE Corrected			Heterogeneity I^2	Metabias P-Value	Excess Significance P-Value
		Voxels	Z Score	P-Value	Voxels	Z Score	P-Value			
Left insula, BA 47	-38, 16, -6	87	4.041	< 0.001				1.966	0.974	1
Right precuneus, BA 30	4, -50, 20	83	3.744	< 0.001				25.647	0.807	0.999
Right anterior thalamic projections	16, 8, 16	57	3.305	< 0.001				1.094	0.973	< 0.001
Left superior frontal gyrus, medial, BA 10	-6, 56, 10	38	2.876	0.002				35.225	0.561	0.998
Right median network, cingulum	8, 38, 2	21	3.186	< 0.001				4.478	0.911	1

eTable 9: Omnibus meta-analysis results: psychosocial treatment subgroup

© 2025 Evohr B et al. *JAMA Network Open*.

Region	MNI Coordinate	Uncorrected			TFCE Corrected			Heterogeneity I^2	Metabias P-Value	Excess Significance P-Value
		Voxels	Z Score	P-Value	Voxels	Z Score	P-Value			
Left inferior occipital gyrus, BA 19	-42, -74, -6	744	5.109	< 0.001	288	5.109	0.004	20.518	0.048	1
Right heschl gyrus, BA 48	50, -8, 6	820	4.576	< 0.001	104	4.576	0.03	6.835	0.459	0.415
Left superior frontal gyrus, medial, BA 10	-8, 60, 0	601	4.953	< 0.001	15	4.953	0.038	0.779	0.82	1
Bilateral posterior cingulate	2, -46, 20	289	3.939	< 0.001				22.652	0.836	0.275
Right superior parietal gyrus, BA 7	28, -62, 52	207	3.884	< 0.001				23.702	0.214	0.774
Left rolandic operculum, BA 48	-54, -2, 10	198	4.026	< 0.001				12.744	0.163	1
Right inferior temporal gyrus, BA 19	44, -72, -8	174	3.894	< 0.001				26.784	0.036	< 0.001
Right inferior network, inferior longitudinal fasciculus	22, -94, 4	148	3.896	< 0.001				0.283	0.822	0.829
Right cortico-spinal projections	6, -22, -6	127	3.935	< 0.001				6.443	0.53	< 0.001
Left inferior parietal (excluding supramarginal and angular) gyri, BA 40	-34, -50, 48	122	3.724	< 0.001				6.698	0.306	0.823
Left hippocampus, BA 20	-26, -18, -18	70	3.6	< 0.001				1.301	0.591	1
Right fusiform gyrus, BA 18	22, -66, -12	62	3.493	< 0.001				0.238	0.651	1

© 2025 Evohr B et al. *JAMA Network Open*.

Region	MNI Coordinate	Uncorrected			TFCE Corrected			Heterogeneity I^2	Metabias P-Value	Excess Significance P-Value
		Voxels	Z Score	P-Value	Voxels	Z Score	P-Value			
Right caudate nucleus	16, 4, 20	57	3.736	< 0.001				2.225	0.927	1
Left middle frontal gyrus, BA 46	-34, 30, 36	16	3.031	0.001				3.66	0.505	1
Left supplementary motor area, BA 6	-4, -2, 68	13	3.021	0.001				4.544	0.382	0.999
Right inferior occipital gyrus, BA 19	38, -86, -2	10	2.896	0.002				9.047	0.972	0.998

I^2 quantifies the proportion of variability in voxel-wise effect sizes that is due to true differences between studies, with higher values (e.g. >50) indicating greater heterogeneity and lower consistency in activation patterns across studies.

The metabias *p*-value is a statistical measure used to detect potential publication bias or small-study effects in a meta-analysis. It assesses whether smaller studies tend to report disproportionately larger effect sizes compared to larger studies, which may suggest selective reporting of positive findings. If the metabias *p*-value is <0.05, this indicates statistically significant evidence of bias, suggesting that the results of the meta-analysis may be influenced by the selective publication of studies with larger or more favorable effects.

The excess significance *p*-value tests whether there are more significant findings across studies than expected given their statistical power, with a *p*-value < 0.05 suggesting potential reporting bias or inflation of effects due to selective publication.

© 2025 Evohr B et al. *JAMA Network Open*.

eFigure 1: Brain activation to drug cues over neutral in omnibus meta-analysis

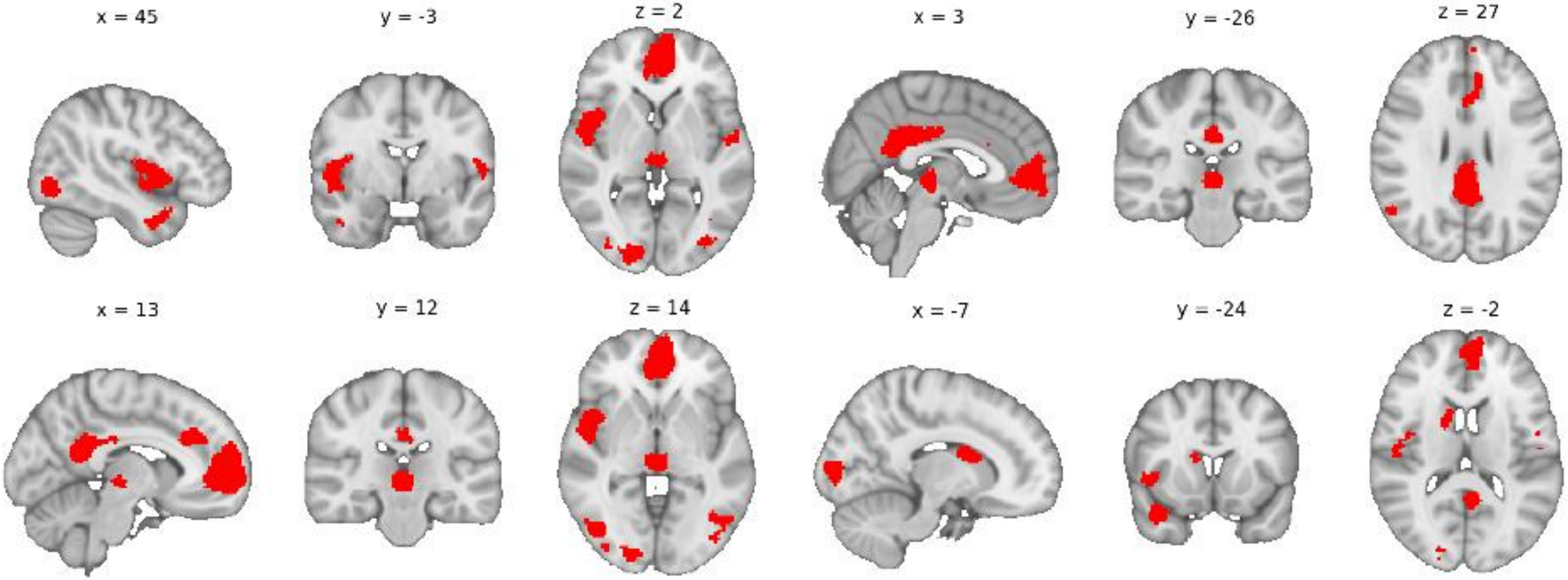

© 2025 Evohr B et al. *JAMA Network Open*.

eFigure 2: Cue reactivity as a predictive biomarker

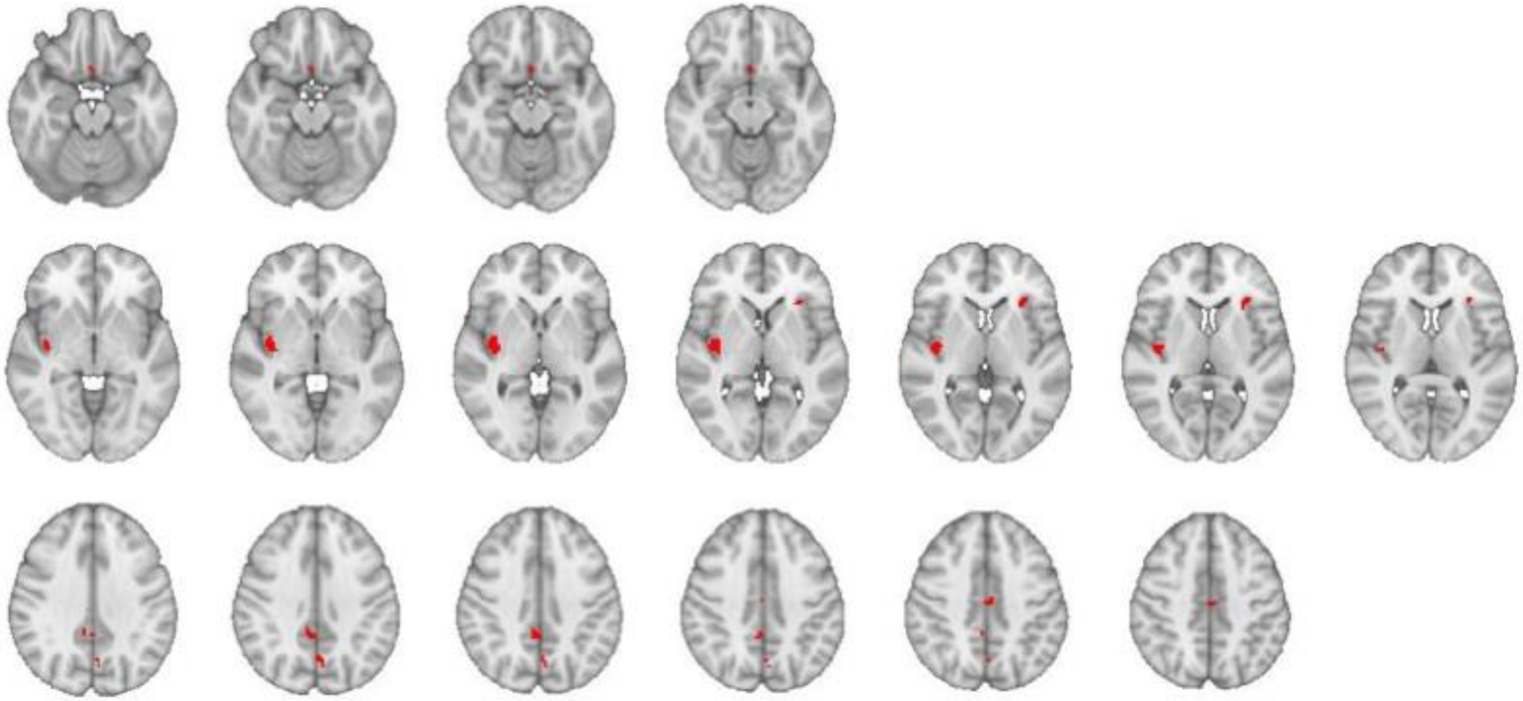

Images represent Z = -18 to Z = - 14, Z = -4 to Z = 8, and Z = 34 to Z = 44 on the axial plane.

© 2025 Evohr B et al. *JAMA Network Open*.

eFigure 3: Cue reactivity as a response biomarker (time)

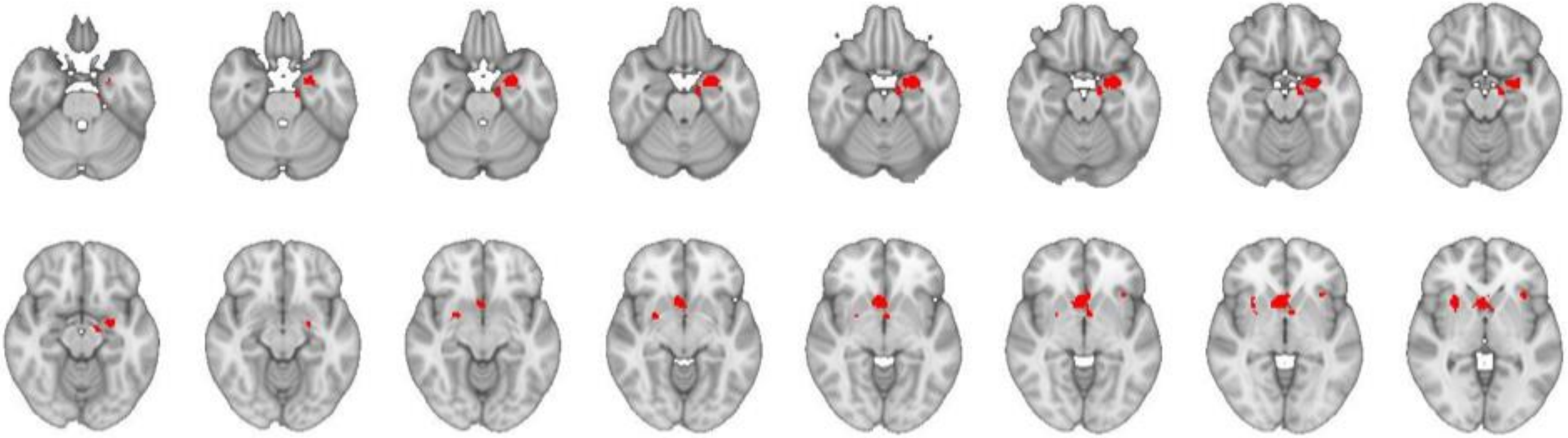

Images represent Z = -28 to Z = -1 on the axial plane.

© 2025 Evohr B et al. *JAMA Network Open*.

eFigure 4: Cue reactivity as a response biomarker (time x treatment)

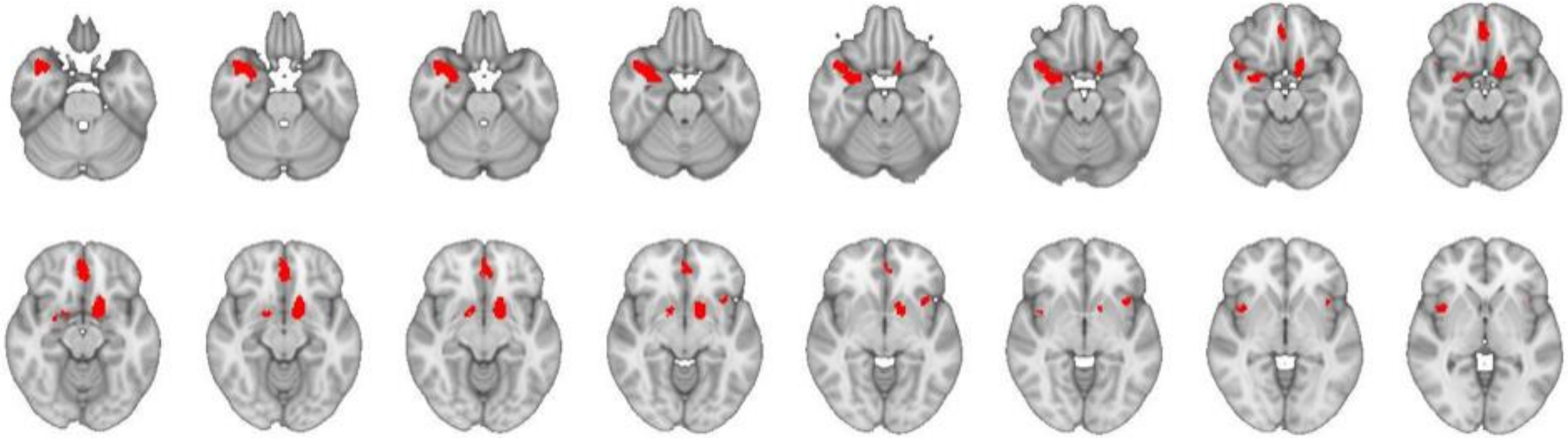

Images represent Z = -28 to Z = -1 on the axial plane.

© 2025 Evohr B et al. *JAMA Network Open*.

eFigure 5: Funnel plots for significant clusters identified in the SDM meta-analysis

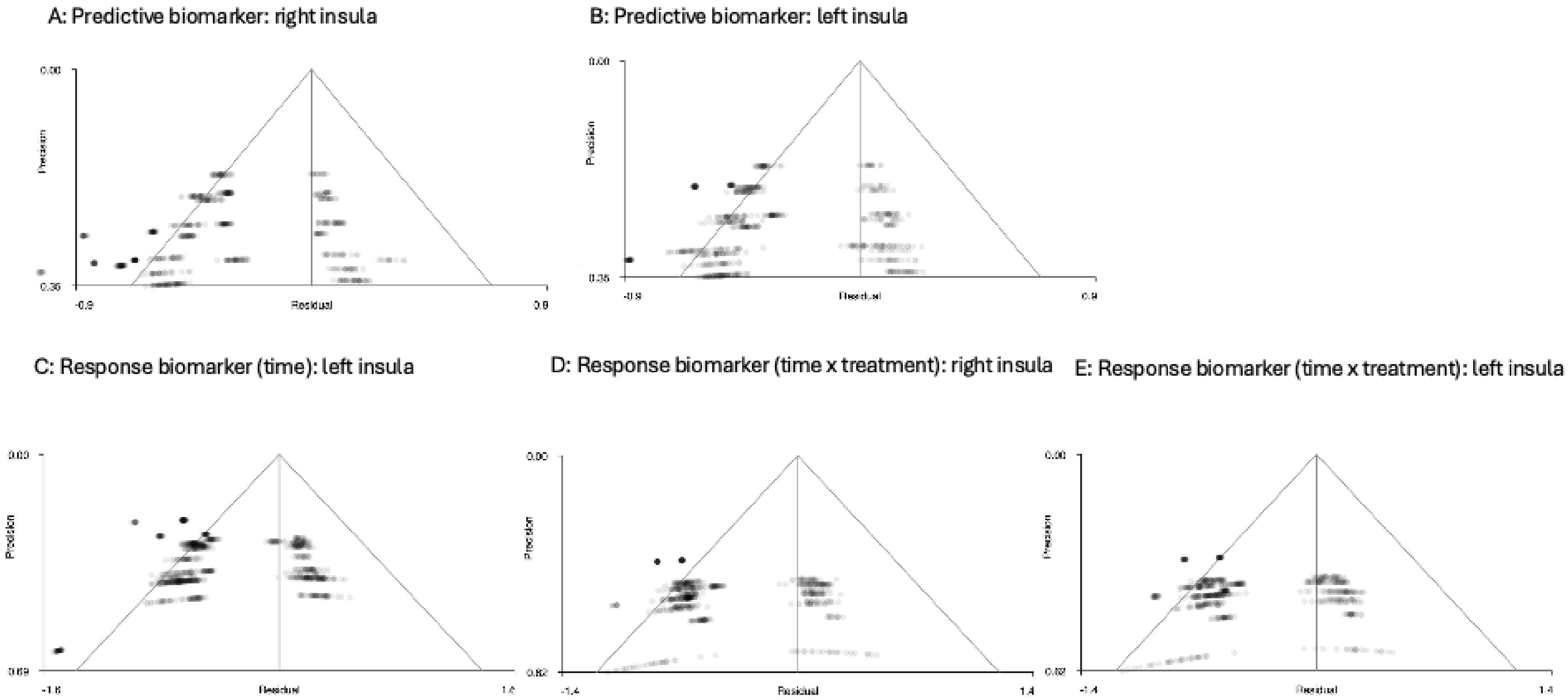

The vertical line denotes residuals of 0, where each study's effect size matches the pooled SDM effect size, and the diagonal lines mark the expected 95% confidence region. Symmetric distributions of points suggest little evidence of publication bias, whereas asymmetry, particularly among smaller studies with greater variance (lower precision), may reflect small-study effects or selective reporting.

© 2025 Evohr B et al. *JAMA Network Open*.
