# Supplementary material for: Cue-Elicited Brain Activity and Treatment Outcomes in Substance Use Disorders: A Meta-Analysis
Source: JAMA Netw Open. 2025 Dec 11;8(12):e2548809. doi: 10.1001/jamanetworkopen.2025.48809 (PMC12699361; doi:10.1001/jamanetworkopen.2025.48809)
Supplement: Supplement 2. — Data Sharing Statement [file jamanetwopen-e2548809-s002.pdf]

Data Sharing Statement

Evohr. Cue-Elicited Brain Activity and Treatment Outcomes in Substance Use Disorders. *JAMA Netw Open*. Published December 11, 2025. doi:10.1001/jamanetworkopen.2025.48809

Data
Data available: Yes
Data types: Data (not involving human participants)
How to access data: The data used in this meta-analysis were extracted from previously published studies. All included coordinates and metadata are available in the Supplementary Materials. Additional analytic scripts, thresholded statistical maps, and methodological details are available upon reasonable request from the corresponding author. No individual participant data were collected or generated for this study.
When available: With publication

Supporting Documents
Document types: Statistical/analytic code
How to access documents: Please send all requests to jgilman1@mgh.harvard.edu
When available: With publication

Additional Information
Who can access the data: This data will be available to researchers whose proposed use of the data has been approved.
Types of analyses: This data will be available to researchers whose proposed use of the data has been approved for further publications.
Mechanisms of data availability: This data will be available to researchers whose proposed use of the data has been approved for further publications with a signed data access agreeement.
